# Termite Mushrooms (*Termitomyces*), a Potential Source of Nutrients and Bioactive Compounds Exhibiting Human Health Benefits: A Review

**DOI:** 10.3390/jof9010112

**Published:** 2023-01-13

**Authors:** Soumitra Paloi, Jaturong Kumla, Barsha Pratiher Paloi, Sirasit Srinuanpan, Supawitch Hoijang, Samantha C. Karunarathna, Krishnendu Acharya, Nakarin Suwannarach, Saisamorn Lumyong

**Affiliations:** 1 Research Center of Microbial Diversity and Sustainable Utilization, Chiang Mai University, Chiang Mai 50200, Thailand; 2 Department of Biology, Faculty of Science, Chiang Mai University, Chiang Mai 50200, Thailand; 3 Department of Chemistry, Faculty of Science, Chiang Mai University, Chiang Mai 50200, Thailand; 4 Center for Yunnan Plateau Biological Resources Protection and Utilization, College of Biological Resource and Food Engineering, Qujing Normal University, Yunnan 655011, China; 5 Molecular and Applied Mycology and Plant Pathology Laboratory, Centre of Advanced Study, Department of Botany, University of Calcutta, 35, Ballygunge Circular Road, Kolkata, West Bengal 700019, India; 6 Academy of Science, The Royal Society of Thailand, Bangkok 10300, Thailand

**Keywords:** basidiomycetous fungi, biological property, edible mushrooms, ethno-medicine, nutritional values

## Abstract

Termite mushrooms have been classified to the genus *Termitomyces*, family *Lyophyllaceae*, order *Agaricales*. These mushrooms form a mutualistic association with termites in the subfamily *Macrotermitinae*. In fact, all *Termitomyces* species are edible and have unique food value attributed to their texture, flavour, nutrient content, and beneficial mediational properties. Additionally, *Termitomyces* have been recognized for their ethno-medicinal importance in various indigenous communities throughout Asia and Africa. Recent studies on *Termitomyces* have indicated that their bioactive compounds have the potential to fight against certain human diseases such as cancer, hyperlipidaemia, gastroduodenal diseases, and Alzheimer’s. Furthermore, they possess various beneficial antioxidant and antimicrobial properties. Moreover, different enzymes produced from *Termitomyces* have the potential to be used in a range of industrial applications. Herein, we present a brief review of the current findings through an overview of recently published literature involving taxonomic updates, diversity, distribution, ethno-medicinal uses, nutritional value, medicinal importance, and industrial implementations of *Termitomyces*, as well as its socioeconomic importance.

## 1. Introduction

As the second largest group of organisms, fungi are estimated to comprise 11.7–13.2 million species; however, to date, only 150,000 fungal species have been fully explored [1,2]. The huge degree of diversity of this organism, along with differing climatic conditions and a wide range of distribution, have all contributed to fungi being recognized as an ultimate source of natural compounds that can have a significant impact on human health, the economy, and the environment [3,4]. Fungi producing fruit bodies are called “macrofungi” or “mushrooms” that are large enough to be observed by the naked eye. They are able to grow either above ground or underground. Mushrooms are distributed throughout the world and play an important role in associations with mycorrhizae, saprotroph, parasites, and insects in various ecosystems. Mueller et al. [5] estimated that there are 53,000 to 110,000 mushroom species in the world. Up to the present time, approximately 14,000 species have been officially described [6]. In 2021, more than 2189 wild edible mushrooms were reported to be from different parts of the world, of which the highest number of edible mushrooms were reported in Asia (1493 species), followed by Europe (629 species), North America (487 species), Africa (351 species), South America (204 species), Central America (100 species), and the Oceanic Region (19 species). Wild edible mushrooms are routinely consumed in many modern-day communities due to their high nutritional value [7,8,9]. Beneficially, mushrooms are known to possess high protein and fiber contents along with various health-promoting nutrients. They are also known to be low in calories and to contain very low amounts of fat and cholesterol [9], as well as being a good source of vitamins (thiamin, riboflavin, cobalamin, ascorbic acid, calciferol, and tocopherol) and essential minerals (iron, phosphor, copper, potassium, and selenium) [10,11]. For example, wild edible mushrooms, *Boletus edulis*, *Cantharellus cibarius*, and *Lactifluus piperatus* contain 80% to 90% moisture, a good amount of protein (2.67–7.39% dw), and low levels of fat (0.18–1.70% dw) [8,11]. Accordingly, differing regions and/or groups of local peoples utilize wild mushrooms differently, i.e., as a popular food source or for their medicinal or nutritional properties and most mushroom species are consumed after boiling or frying, i.e., cooked condition, but sometimes very few are consumed as raw, for example *Laetiporus sulphureus* in Cameroon [7].

Interestingly, members of the genus *Termitomyces* commonly grow in association with termites. They have unique importance as a food source and also hold promise in the development of nutritional supplements and for their ethno-medicinal prospects, as well as in the socio-economic development of local communities [12,13,14,15]. Historically this genus has not only been regarded for its edibility, the Yoruba people of Nigeria have also used this mushroom in their mythological practices [16]. However, the potential taxonomic development, nutritional prospects, mediational importance, and socio-economic significance of this genus have not yet been fully investigated when compared with other wild edible mushroom genera, e.g., *Amanita*, *Cantharellus*, *Lactarius*, *Lentinus*, and *Russula*. In this review, we have summarized the current findings related to the taxonomic updates, species diversity, distribution, and the potential utilization of the *Termitomyces* species as an alternative supplementary food source as well as for its potential social-economic and industrial development in future. 

## 2. Overview of Taxonomic Implementation

The genus *Termitomyces* was established by R. Heim in 1942 [17]. Soon after the discovery of this genus, Singer [18] recognized a new genus, namely *Podabrella,* and *P. microcarpa* (synonym: *Termitomyces microcarpus*) was proposed as the type species. *Podabrella* species was placed in the subgenus *Praetemitomyces*. However, this classification has not been accepted by Heim [19] and Pegler [20] as they have held back all *Podabrella* species in the genus *Termitomyces* due to their morphological similarity with other species belonging to the genus *Termitomyces* and their association with termites [21]. The early identification and classification of *Termitomyces* has been broadly studied based on comparisons of relevant morphological characteristics. The first detailed study of this genus was summarized by Heim in his monograph “Termite Et Champgnon” [19] of *Termitomyces* species from Africa and Asia. Later, Jiilich [22] elevated this genus to the family level, namely *Termitomycetaceae*, together with *Amanitaceae* and *Torrendiaceae* under the order *Amanitales*. Pegler [23] chose to accommodate this genus within the family *Pluteaceae* due to its morphological similarity with *Pluteaceae* (free and crowded lamella, pink spores print, glutted basidiospores, and hymeneal cystidia) [24]. However, the morphological identification of *Termitomyces* has been limited due to the high degree of phenotypic variability that exists across a wide range of geographic distribution, varying environmental conditions, and the fact that the developmental stage may make morphological identification difficult among other closely related species. Thus, it is essential to identify *Termitomyces* species by applying a DNA-based analysis of its molecular data.

In 2002, the molecular phylogeny along with the morphological characteristics was used for a more prominent identification of the *Termitomyces* species. Rouland-Lefevre et al. [25] used 15 *Termitomyces* samples to establish any relevant molecular relationships based on the internal transcribed spacers (ITS) of the nuclear ribosomal DNA region. Some molecular studies focusing on the host specificity of termites and fungal associations were conducted by Aanen et al. [26] and employed the large subunit (nrLSU) region of the nuclear ribosomal DNA (nrLSU) and the mitochondrial small subunit (mtSSU) region for molecular identification. Taprab et al. [27] combined the ITS and nrLSU regions for effective identification of the *Termitomyces* species. Molecular phylogenetic analysis has revealed that the genus *Termitomyces* forms a monophyletic clade in the family *Lyophyllaceae*, order Agaricales [25,28]. Frøslev et al. [29], also indicated that *Termitomyces* and *Sinotermitomyces* are actually congeneric based on nrLSU and mtSSU sequence analysis [25]. However, the most significant phylogenetic study on *Termitomyces* based on an analysis of nrLSU and mtSSU sequences was provided by Mossebo et al. [30], wherein the *Termitomyces* species was found to include 74 strains belonging to 28 taxa. Sawhasan et al. [31] reported nine known *Termitomyces* species distributed throughout Thailand using ITS sequences. Another molecular study conducted in Africa determined that ITS sequences could be used for accurate *Termitomyces* identification. Recently, may new species have also been identified and proposed based on morpho-molecular taxonomic techniques. Accordingly, Mossebo et al. [30] reported a new combination species, namely *T. brunneopileatus* from Cameroon, based on nrLSU and mtSSU sequences. Ye et al. [32] identified *T. fragilis* from China based on ITS sequence. Tang et al. [33] identified *T. floccosus* and *T. upsilocystidiatus* from China and Thailand based on combined nrLSU and mtSSU regions. Seelan et al. [34] identified *T. gilvus* from Malaysia based on nrLSU and mtSSU sequences. Izhar et al. [35] identified *T. sheikhupurensis* from Pakistan based on a combination of ITS and nrLSU sequences. Additionally, *T. cryptogamus* was described from Africa based on a phylogenetic analysis of the ITS sequence [36]. Therefore, it is essential to be able to identify *Termitomyces* by coordinating both morphological characteristics and molecular approaches through the phylogenetic analyses of ITS, nrLSU, and mtSSU sequences.

## 3. Species Diversity and Distribution

**Figure 1:**
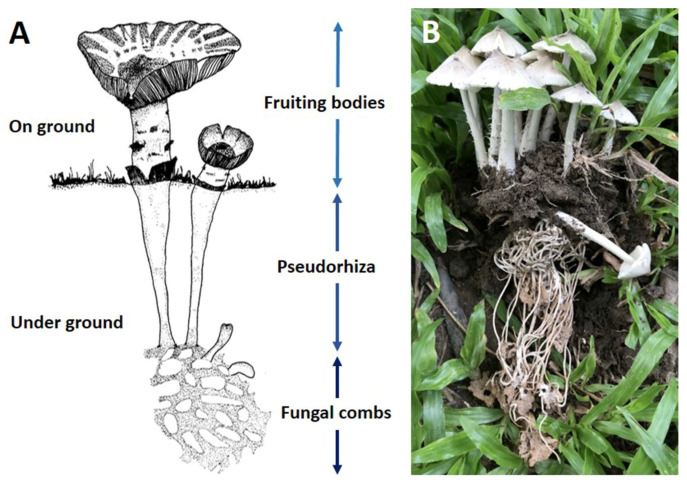
Illustration (**A**) and field photograph (**B**) of fruitbodies of the *Termitomyces* with different parts. Photo credit: Paloi, S. and Kumla, J.

**Figure 2:**
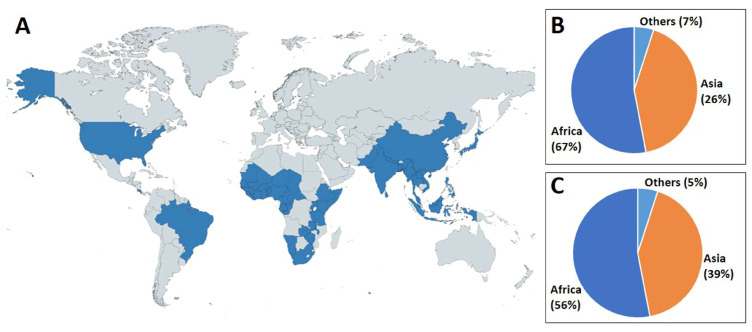
Overview of worldwide distribution of *Termitomyces* species (**A**) (highlighted as blue color, the map was created using MapChart [69]); type species discovery (**B**) and distribution of species (**C**).

*Termitomyces* grows in association with fungal-growing termites belonging to the subfamily *Macrotermitinae*. It is frequently found in the ecosystems of tropical regions [37]. More than 330 species of termites, especially those classified within the genus *Odontotermes*, *Macrotermes*, and *Microtermes*, have been reported to be associated with the cultivation of *Termitomyces* [26,38]. The mutualistic symbiosis between *Termitomyces* and termites was established at least 31 million years ago [39], where termites provided a constant environment for fungal growth as well as to help in the dispersal of spores. In turn, *Termitomyces* provide food for the termites [40]. Generally, termites cultivate *Termitomyces* mycelia on special structures within their nests called “fungal combs”. Fruiting bodies of *Termitomyces* develop from these fungal combs (Figure 1) when the environment is favorable. The seasonal fructification (especially during the rainy season) of *Termitomyces* is restricted to the paleotropical region (African, Asian, and the Pacific Island region), but it is also found in America (Figure 2A) [37]. During the period from 1945 to 1990, many *Termitomyces* species have been found in Africa and Asia. Otieno [41] reported on the identification of five new species with 10 known species being from East Africa. Another study conducted by Pegler and Rayner [42] reported that 11 species were from the same region, while some previous studies [21,43,44] identified seven and five species, respectively, from South Africa. Furthermore, Alasoadura [45] identified six species from Nigeria, and Moriss [46] reported on eight species from Malawi. One new species, *Termitomyces titanicus*, was reported to be from Zambia along with 10 other known species [47,48]. 

Taxonomic treatments of the genus *Termitomyces* in Asia were mainly conducted by several previous studies [49,50,51,52,53,54,55,56,57,58]. *Termitomyces* species were reportedly from India and 22 taxa were reported to be from Asia. The type revision of three Indian *Termitomyces* species was conducted by Tang et al. [59] and Pegler and Vanhaecke [24] in South East Asia. They reported on the existence of 14 *Termitomyces* species from China, India, Malaysia, Philippines, Thailand, etc. [60,61,62,63,64], while Tang et al. [65] reported that many *Termitomyces* species were collected from different parts of India and China. Sawhasan et al. [31] and Jannual et al. [66] have also provided distributional records of several *Termitomyces* species from Thailand, while Kobayashi et al. [67] identified several species from Japan. Currently, worldwide distribution of *Termitomyces* comprises 58 species [68]. The list of *Termitomyces* species and their known range of distribution are summarized in Table 1.

**Table 1:** List of *Termitomyces* species, origin, and their known distributions.

*Termitomyces* Species	Origin	Known Distribution	References
*T. acriumbonatus* Usman and Khalid	Pakistan	Pakistan	[70]
*T. albidolaevis* Dhanch., J.C. Bhatt and S.K. Pant	India	India	[57]
*T. albidus* (Singer) L.D. Gómez	Costa Rica	Costa Rica	[71]
*T. albus* (Peck) L.D. Gómez	New York	New York and USA	[71]
*T. aurantiacus* (R. Heim) R. Heim	Central Africa	Tanzania, Malawi, Ethiopia, Cameroon, Ivory Coast, Thailand, China, Nepal, and Malaysia	[19,24,29,72,73,74,75,76,77,78]
*T. badius* Otieno	Kenya	Kenya, India, Nepal, and Myanmar	[76,79,80,81]
*T. biyi* Otieno	Kenya	Kenya	[41]
*T. brunneopileatus* Mossebo and Essouman	Cameroon	Cameroon	[30]
*T. bulborhizus* T.Z. Wei, Y.J. Yao, Bo Wang and Pegler	China	China; Thailand, Philippines, India, Myanmar, Laos, and Nigeria	[31,62,81,82,83,84,85]
*T. cartilagineus* (Berk.) R. Heim	Sri Lanka	Sri Lanka	[86]
*T. citriophyllus* R. Heim	Guinea	Guinea	[17]
*T. clypeatus* R. Heim	Congo	Congo, Ethiopia, Burundi, South Africa, Tanzania, Nigeria, Cameroon, Kenya, Uganda, Ivory Coast, Ghana, Thailand, Japan, Malaysia, Philippines, India, China, Myanmar, Nepal, Laos, and Bangladesh	[24,26,44,64,66,72,75,78,81,82,85,87,88,89,90,91,92,93,94,95]
*T. congolensis* (Beeli) Singer	Congo	Congo	[96]
*T. cryptogamus* van de Peppel	South Africa	South Africa	[36]
*T. dominicalensis* L.D. Gómez	Costa Rica	Costa Rica	[71]
*T. entolomoides* R. Heim	Congo	Congo, China, Thailand, India, and Malaysia	[31,64,87,97,98]
*T. epipolius* (Singer) L.D. Gómez	Brazil	Brazil	[71]
*T. eurrhizus* (Berk.) R. Heim	Sri Lanka	Uganda, Tanzania, Zambia, Kenya, Malawi, Ethiopia, Sri Lanka, China, Japan, India, Philippines, Nepal, Thailand, Malaysia, and Myanmar	[17,58,64,67,75,81,94,99,100,101,102,103,104]
*T. floccosus* S.M. Tang, Raspé and S.H. Li	Thailand	Thailand	[33]
*T. fragilis* L. Ye, Karun, J.C. Xu, K.D. Hyde and Mortimer	China	China	[32]
*T. fuliginosus* R. Heim	Guinea	Guinea, Ivory Coast, Thailand, and Vietnam	[17,37,66]
*T. gilvus* C.S. Yee and J.S. Seelan	Malaysia	Malaysia	[34]
*T. globulus* R. Heim and Gooss.-Font.	Congo	Congo, Ghana, Nigeria, Uganda, Cameroon, Kenya, Thailand, China, Indonesia, India, and Nepal	[14,16,20,24,64,87,91,98,99,105,106]
*T. griseiumbo* Mossebo	Cameroon	Cameroon	[107]
*T. heimii* Natarajan	India	Kenya, Ivory Coast, India, Malaysia, Nepal, Pakistan, China, Thailand, Myanmar, and Bangladesh	[14,24,52,64,66,81,94,95,108,109]
*T. indicus* Natarajan	India	India	[49]
*T. infundibuliformis* Mossebo	Cameroon	Cameroon	[110]
*T. lanatus* R. Heim	Central African	Central African	[19]
*T. le-testui* (Pat.) R. Heim	Congo	Congo, Tanzania, Cameroon, Zimbabwe, Ivory Coast, Ethiopia, Malawi, Nepal, China, and India	[17,29,30,37,75,76,111]
*T. magoyensis* Otieno	Kenya	Kenya	[41]
*T. mammiformis* R. Heim	Guinea	Guinea, Burundi, Zambia, China, and India	[17,29,64,72,112]
*T. mboudaeinus* Mossebo	Cameroon	Cameroon	[107]
*T. mbuzi* Härkönen and Niemelä	Tanzania	Tanzania	[113]
*T. medius* R. Heim and Grassé	French Equatorial Africa	French Equatorial Africa, Cameroon, Ivory Coast, and India	[29,37,114,115]
*T. meipengianus* (M. Zang and D.Z. Zhang) P.M. Kirk	Yunnan	China	[116]
*T. microcarpus* (Berk. and Broome) R. Heim	Sri Lanka	Uganda, Zimbabwe, Cameroon, Tanzania, South Africa, Malawi, Ethiopia, Japan, Ghana, Nepal, Sri Lanka, Malaysia, India, Pakistan, Philippines, China, Thailand, and Laos	[14,24,29,31,64,67,74,85,86,91,117,118]
*T. narobiensis* Otieno	Kenya	Kenya	[41]
*T. orientalis* R. Heim	Kenya	Kenya	[19]
*T. perforans* R. Heim	Central African	Central African, Nigeria, Thailand, and India	[19,119,120,121]
*T. poliomphax* (Singer) L.D. Gómez	Brazil	Brazil	[71]
*T. rabuorii* Otieno	Kenya	Kenya	[41]
*T. radicatus* Natarajan	India	India, Thailand, and Malaysia	[31,50,98]
*T. reticulatus* Van der Westh. and Eicker	South Africa	South Africa, Cameroon, and India	[21,122,123]
*T. robustus* (Beeli) R. Heim	Congo	Congo, Uganda, Burundi, Tanzania, Ethiopia, Ivory Coast, Philippines, Nigeria, Ghana, Nepal, and India	[14,26,29,42,75,78,87,91,124,125,126]
*T. sagittiformis* (Kalchbr. and Cooke) D.A. Reid	South Africa	South Africa, Tanzania, and India	[72,127]
*T. schimperi* (Pat.) R. Heim	Africa	Ethiopia, Tanzania, Kenya, Namibia, Zambia, Malawi, Zimbabwe, Ghana, Ivory Coast, Myanmar, West Africa, India, and Nepal	[14,21,29,42,91,122,128,129,130]
*T. sheikhupurensis* Izhar, Khalid and H. Bashir	Pakistan	Pakistan	[35]
*T. singidensis* Saarim. and Härk.	Tanzania	Tanzania	[131]
*T. songolarum* (Courtec.) Furneaux	Congo	Congo	[132]
*T. spiniformis* R. Heim	Central African	Central African	[19]
*T. striatus* (Beeli) R. Heim	Sierra Leone	Sierra Leone, Kenya, Nigeria, Malawi, Burundi, Cameroon, Ivory Coast, Uganda, Congo, South Africa, Tanzania, China, Malaysia, India, Pakistan, Philippines, Thailand, and Nepal	[14,17,21,24,30,37,40,64,66,72,94,133,134,135]
*T. subclypeatus* Mossebo	Cameroon	Cameroon	[107]
*T. subhyalinus* Moncalvo, Vilgalys, Redhead et al.	Africa	Africa	[136]
*T. subumkowaan* Mossebo	Cameroon	Cameroon	[107]
*T. titanicus* Pegler and Piearce	Zambia	Zambia, Tanzania, Cameroon, Burundi, and South Africa	[47,72,137,138,139]
*T. tylerianus* Otieno	Kenya	Kenya, Tanzania, China, and India	[41,64,140,141]
*T. umkowaan* (Cooke and Massee) D.A. Reid	South Africa	South Africa, Tanzania, Cameroon, Kenya, and Ivory Coast, India, Nepal, Pakistan, and Indonesia	[14,21,78,142,143,144,145,146,147]
*T. upsilocystidiatus* S.M. Tang, Raspé and K.D. Hyde	China	China	[33]

Most type *Termitomyces* species have been discovered in Africa (67%), followed by Asia (26%), and various other continents (7%) (Figure 2B). Accordingly, the greatest degree of species distribution was recorded in Africa (56%), followed by Asia (39%), and other continents (5%) (Figure 2C). Some widely distributed and common *Termitomyces* include *T. aurantiacus*, *T. bulborhizus*, *T. clypeatus*, *T. eurrhizus*, *T. heimii*, *T. medius*, *T. microcarpus*, *T. schimperi*, and *T. striatus*, all of which are found in different parts of Africa and Asia (Table 1). On the other hand, *T. acriumbonatus*, *T. albidolaevis*, *T. cartilagineus*, *T. floccosus*, *T. fragilis*, *T. gilvus*, *T. griseiumbo*, *T. indicus*, *T. meipengianus*, *T. radicatus*, *T. sheikhupurensis*, *T. singidensis*, and *T. upsilocystidiatus* restricted in Asian countries.

## 4. Edibility and Socio-Economic Impact

Mushrooms have extensively been used as a food source for thousands of years due to their unique flavor and beneficial food value [6,7]. Currently, mushrooms are being used as functional food for the prevention of several human diseases [10,148,149]. Termite mushrooms are known for their unique taste and flavor, and are particularly abundant in Africa and Asia [34,46]. Almost all species of *Termitomyces* are edible; however, *T. titanicus* is the world’s largest edible mushroom. It grows abundantly in West Africa as well as Zambia where it is frequently consumed by local people [46,150]. The main reason for its popularity is its nutritional value and beneficial medicinal properties [151,152,153,154].

**Figure 3:**
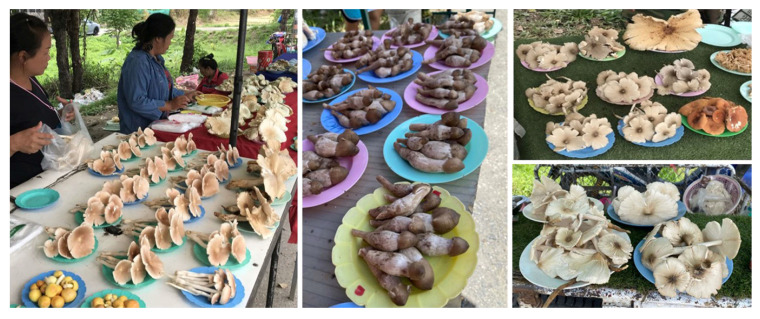
Some *Termitomyces* species are sold in the Thai local and roadside markets. Photo credit: Suwannarach, N.

Throughout Asia, ethnic and native people routinely consume *Termitomyces* during the annual monsoon. It is commonly available at road-side stalls as well as in city markets [12,155,156]. Members of the genus *Termitomyces* are primarily consumed by Indian, Chinese, Laos, and Nepalese people but are also frequently consumed by the populations of a variety of other countries including Thailand and Malaysia. However, in India, several species of *Termitomyces* are consumed in different states including *T. badius*, *T. clypeatus*, *T. eurrhizus*, *T. heimii*, *T. mammiformis*, *T. medius*, *T. microcarpus*, *T. radicatus*, *T. reticulatus*, *T. schimperi*, *T. striatus*, and *T. globulus* [12,58,157,158,159]. Some of these species are also available in local markets with the price ranging between 0.50 and 2.45USD/kg depending upon the region of the point of sale [12,155]. In China, *Termitomyces* are locally known as “Jizong” (chicken mushrooms) and “Yizong” (ant planted mushrooms) [32]. Several species of this genus are also famous for their edibility including *T. microcarpus*, *T. aurantiacus*, *T. bulborhizus*, *T. eurrhizus*, *T. globulus*, and *T. fragilis* [32,160]. According to Wei et al. [64], the *Termitomyces* species was sold on the market at 27.96 USD/kg in 2006, which was quite high. The diversity of edible *Termitomyces* is also quite high in Nepal, where local and ethnic people consume different species of *Termitomyces* including *T. microcarpus*, *T. mammiformis*, *T. heimii*, *T. clypeatus*, *T. eurhizus*, *T. striatus*, and *T. aurantiacus* [76,92,102,161]. Aryal and Budathoki [14] have indicated that nineteen species of *Termitomyces* are commonly consumed in different parts of Nepal. Specifically, *Termitomyces heimii* is sold in markets throughout Nepal at 2.1 to 2.9 USD/kg [161]. “Hed Khone” is the common name for *Termitomyces* in Thailand [162]. *Termitomyces clypeatus*, *T. fuliginosus*, and *T. globulus* have been reported as edible species [163,164] and *T. clypeatus* can be found in markets located in Sakon NaKhon Province selling for around 6.98 to 8.38 USD/kg (Figure 3) [163]. In Malaysia, *Termitomyces* is known by several names such as “Cendawan busut”, “Cendawan meluku”, “Cendawan susu pelanduk”, “Cendawan anai-anai”, “Cendawan guruh”, “Kulat tahun”, “Cendawan Tali”, or “Kulat Taun” [34,165,166,167]. *Termitomyces clypeatus*, *T. eurhizus*, and *T. heimii* are common edible mushrooms that are indigenous to the Malay Peninsula [34,165,167]. A few varieties of *Termitomyces* are famous in Laos for their edibility including *Termitomyces* aff. *aurantiacus* (local name: Phuak tab fai), *T. bulborhizus* (local name: Pouak tam fan), *T. clypeatus* (local ame: Pouak jik), *T. eurrhizus* (het khon kao), *T. fuliginosus*, *T. heimii* (het pouak) *Termitomyces* aff. *heimii* (local name: Pouak tap kan yao), and *T. microcarpus* (local name: Poauk kai noi), all of which are available in local markets [85,168]. Two *Termitomyces* species, e.g., *T. eurrhizus* and *T. microcarpus* frequently consumed in Sri Lanka [169,170].

In Africa, *Termitomyces* is also widely prized for its edibility. Accordingly, near about seven *Termitomyces* species, e.g., *T. letestui*, *T. mammiformis*, *T. microcarpus*, *T. robustus*, *T. schimperi*, *T. striatus*, and *T. titanicus*, are consumed in Burundi [171,172], whereas only four species are consumed in Rwanda [171,172]. Notably, *T. microcarpus*, *T. robustus* can easily be found in markets in Rwanda during rainy seasons. In Namibia, only one species of *Termitomyces*, e.g., *T. schimperi*, is routinely consumed by local people. It is locally known as “omajowa”, while three other species have been found in this region: *T. umkowaani*, *T. microcarpus*, and *T. tyleranus*. These have also been reported as edible mushrooms in other parts of Africa [15].

Interestingly, *T. heimii*, *T. medius*, *T. letestui*, *Termitomyces* cf. *eurhizus*, and *T. fuliginosus* are widely consumed in Côte d’Ivoire [13,109]. *Termitomyces globulus*, *T. aurantiacus*, *T. mboudaeina*, *T. clypeatus*, *T. striatus*, *T. macrocarpus*, *T. schimperi*, and *T. mammiformis* are well regarded in Cameroon for their edibility [93,173,174]. Nigerian people also routinely consume *Termitomyces* in their diets in the form of *T. mammiformis*, *T. robustus*, *T. clypeatus*, *T. striatus*, *T. globulus*, and *T. microcarpus* [90,151,175,176]. The native people of Menge District, Ethiopia consume several *Termitomyces* species as food, e.g., *T. clypeatus*, *T. eurhizus*, *T. letestui*, *T. microcarpus*, *T. schimperi*, *T. robustus*, *T. striatus*, and *T. umkowaanii* [118]. The populations of other African countries, namely Kenia, Sudan, Tanzania, Congo, and Uganda, also consume *Termitomyces* as food [156,177,178,179,180]. Consumption of *Termitomyces* varies depending on the region. *Termitomyces* mushrooms are typically eaten after being cooked. For example, Burundi peoples use *T. robustus* and *T. striatus* to make Steak Ikinyinu [172]. In Thailand, *Termitomyces* species are frequently used to make spicy mushroom salad and mushroom soup. According to the different previous reports, *Termitomyces* can be preserved by drying and brining processes [162,172]. 

The development of non-wood forest products is the primary income source for several ethnic groups in different regions of the world [181]. Many ethnic groups of people collect different non-wood forest products (for example: honey, wild fruit, and edible mushrooms) for resale in the marketplace as a way of earning income [181]. The socio-economic development of products incorporating wild edible mushrooms is a traditional practice among ethnic societies in Asia and Africa [182]. For example, the Benna and Hehe ethnic groups of Tanzania collect 1000–1500 kg wild mushrooms per season and consequently earn 500 to 650 USD [156]. However, *Termitomyces* is one of the most famous wild edible mushrooms that has contributed to the socio-economic development of this country due to its high market value. For example, certain tribal peoples (Santals, Bhumij, Lodha, Munda, and others) from West Bengal, India sell *Termitomyces* at local village markets or in small city markets and earn 0.5 to 2.5 USD/kg [12,155]. Manna and Roy [155] have estimated that 9.83% and 10.29% of the total annual income of a Santal family can come from the harvesting of wild mushrooms of the Choupahari and Gonpur forest areas, respectively.

## 5. Ethnomedicinal Importance

Folk medicine has long been a traditional practice and a key cultural element of ethnic communities all over the world [183]. This type of practice can involve plants and plant parts, as well as also other harvestable organisms including mushrooms [184,185]. The ethnomedicinal importance of different *Termitomyces* species are summarized in Table 2. Different ethnic groups have their own priorities in the way they choose to utilize natural resources, for example some east Asian countries (China and Japan) have well-documented their traditional knowledge of mushrooms and have also found ways to use this knowledge in the present, but several countries have not retained this type of knowledge in a well-documented form [186,187]. However, several members of the genus *Termitomyces* have been recognized for their ethnomedicinal importance to different ethnic groups and countries [14,72]. For example, *T. microcarpus* is widely distributed across certain continents (Asia and Africa) and can be employed in different ethnomedicinal applications in differing locations. In Nigeria (especially among the Yoruba people) this species is used to treat gonorrhea [16,184], while in India it is used to treat fevers, colds, and fungal infections [188]. Furthermore, the native people of Tanzania and Nepal use it to boost the immunosystem and consume it in the form of a tonic as an energy stimulant, respectively [14,72]. The native people of the Kilum-Ijim forest area (Cameroon) use this mushroom to strengthen bones in children and to treat fever [135]. However, a valuable publication by Aryal and Budathoki [14] reported on the ethnomedicinal importance of *Termitomyces* in Nepal and described nineteen *Termitomyces* species consumed by local and ethnic people in the treatment of several diseases [14].

**Table 2:** Ethno-medicinal importance of different *Termitomyces* species.

*Termitomyces* Species	Ethno-Medicinal Importance	Country	Ethnic Group	References
*T. aurantiacus*	Used as tonic in fever	Nepal	NR	[14]
*T. badius*	Used for the constipation, Laziness, Indolence, and inactiveness	Nepal	NR	[14]
*T. clypeatus*	Used for the treatment of pox	India	Santal	[189,190]
	Used for the remedy of measles, yellow fevers	Nepal	NR	[14]
	Treating constipation and gastritis in adults, and highly recommended for underweight children	Ethiopia	NR	[118]
*T. eurrhizus*	Used for the treatments of rheumatism, diarrhea, and lowering high blood pressure	India	Santal, Kolha, Munda, Khadia, Bhumija, Bhuyan, Bathudi, Ho, Kudumi, and Mankdias	[191]
	Used for skin diseases with mixing the herb (Cynodon doctylon)	Nepal	NR	[14]
	Used in fever and measles	India	NR	[192]
	Used for recovery chicken pox	India	Santal	[190]
*T. globulus*	Used for wound healing	Nepal	NR	[14]
*T. heimii*	Used in treatment for fever, cold, and fungal infections	India	NR	[188]
	Used in blood tonics during wound healing and blood coagulation	India	NR	[193]
	Syrup is used for Jaundice and diarrhea	Nepal	NR	[14]
*T. letestui*	Used in remedy of inappetence, abdominal disorder, Indigestion, and stomachache	Nepal	NR	[14]
*T. mammiformis*	Used in abdominal discomfort, cough and whooping cough	India	Mokokchung	[194]
	Used in increase body strengthen	Nepal	NR	[14]
*T. microcarpus*	Used in Bone strengthening for children and Fever	Cameroon	local peoples from Kilum-Ijim forest area	[135]
	Used in treatment for fever, cold, and fungal infections	India	NR	[188]
	Used in gonorrhea treatment	Nigeria	Yoruba	[16,187]
	Used for boosting immune system	Tanzania	NR	[72]
	Tonic for stimulating power	Nepal	NR	[14]
	Used in constipation, gastritis in adults, and highly recommended for underweight children	Ethiopia	NR	[118]
*T. reticulatus*	Used in rheumatism and lowering high blood pressure	India	Kharia, Mankidi, Santal, Kolha, Munda, Bhumija, Bhuyan, Bathudi, Ho, Kudumi, Mankidia and Birhor	[186]
*T. robustus*	Used in anemia and high blood pressure	Nigeria	NR	[195]
	Used in constipation, laziness, indolence, and inactiveness	Nepal	NR	[14]
	Used in Maagun	Nigeria	Yoruba	[16]
*T. schimperi*	Used in cut wound, and skin diseases	Nepal	NR	[14]
*T. tyleranus*	Used in chicken pox	India	Dangi	[140]
*T. umkowaan*	Used in mouthwash for buccal cavity infection, and arthritics pain	Nepal	NR	[14]

NR = Not reported.

## 6. Nutritional Prospects

Fruiting bodies of the *Termitomyces* species are known to offer a significant nutritional value to humans [174,196,197]. According to various scientific investigations on their proximate composition, several *Termitomyces* species regarded as a source of nutrition for humans because of their containing of protein, carbohydrates, and dietary fiber [109,173,174,196,197]. Some examples of the proximate compositions of different *Termitomyces* are presented in Table 3. Additionally, *T. eurrhizus*, *T. microcarpus*, *T. robustus*, *T. striatus*, and *T. umkowaan* are known to contain a number of beneficial minerals (including sodium, potassium, calcium, magnesium, zinc, copper, iron, phosphorus, and manganese) and vitamins (vitamin A, thiamine, ascorbic acid, tocopherol, and others) [174,197] (Table 3). The *Termitomyces* species is also known to contain different types of amino acids, e.g., histidine, isoleucine, leucine, lysine, methionine, phenylalanine, threonine, valine, arginine, aspartic acid, serine, glutamic acid, proline, glycine, alanine, cysteine, and tyrosine [196]. According to Karun et al. [147], the uncooked *T. umkowaan* has greater crude fiber, ash, and minerals compared to cooked conditions; however, there are no significant differences variations in crude protein, fat, and carbohydrate.

**Table 3:** Proximate compositions of different *Termitomyces* species.

*Termitomyces* Species	Proximate Composition (% Dry Weight)					Others (mg/100 g Dry Weight)	References
	Carbohydrate	Protein	Fats	Fiber	Ash		
*T. aurantiacus*	46.44	16.62	2.70	24.68	9.56	NR	[173]
*T. clypeatus*	27.67	26.34	7.90	35.15	2.94	NR	[163]
*T. eurrhizus*	NR	29.40	6.27	26.64	11.52	Calcium (100), Iron (50), Magnesium (160), and Potassium (2360)	[197]
*T. heimii*	47.66	23.75	3.58	6.02	7.40	NR	[109]
*T. letestui*	43.65	19.13	5.14	23.13	8.45	NR	[173]
*T. mammiformis*	47.56	15.07	5.42	17.56	14.39	NR	[173]
*T. mboudaeina*	45.33	17.26	2.63	24.10	10.68	NR	[173]
*T. microcarpus*	44.23	30.69	2.17	11.60	11.30	Calcium (37.47), Magnesium (39.03), Phosphorus (898.17), Potassium (1112.76), and Sodium (12.91)	[174]
*T. robustus*	24.90	42.77	6.76	4.07	10.45	Calcium (60), Copper (0.90), Iron (2.70), Magnesium (106), Phosphorus (30.80), Potassium (1460), Sodium (270), and Zinc (81)	[196]
*T. schimperi*	57.42	14.48	2.64	20.29	5.17	NR	[173]
*T. striatus*	46.82	21.76	2.40	16.70	12.33	Calcium (26.39), Magnesium (28.47), Phosphorus (739.06), Potassium (1450.44), and Sodium (12.31)	[174]
*T. titanicus*	58.08	27.22	7.90	NR	6.80	NR	[198]
*T. umkowaan*	45.26	18.89	4.50	15.73	2.58	Calcium (15.60), Copper (0.15), Iron (6.80), Magnesium (25.10), Phosphorus (63.73), Potassium (75.40), Sodium (26.20), Zinc (2.20)	[143]

NR = Not reported.

## 7. Bioactive Compounds

### 7.1. Phenolic Compounds

Phenolic compounds are the most abundant secondary metabolite found in several varieties of mushrooms [199,200,201]. The common chemical structure of the phenolic compounds comprise one or more than one hydroxyl substituents attached to an aromatic ring. Phenolic acids, flavonoids, lignans, stilbenes, and tannins are the major phenolic groups [201]. Phenolic compounds are known to have a great impact on various biological activities, e.g., antimicrobial, antioxidant, and anti-inflammatory properties [202]. However, wild macrofungi can be a good alternative source of phenolic compounds. Many edible and medicinally important macrofungi contain different types of phenolic compounds that may have a great benefit to human health [9,203]. Members of the genus *Termitomyces* possess a huge number of phenolic compounds and are well-documented to have originated from different corners of the world [196,204,205]. Most of these studies have been undertaken to measure the total amount or the presence/absence of different phenolic compounds, e.g., flavonoids, lignans, and stilbenes. The *Termitomyces* species is known to contain different phenolic compounds including gallic acid, chlorogenic acid, caffeic acid, ellagic acid, catechins, epicatechins, rutin, isoquercitrin, quercitrin, quercetin, and kaempferol (Table 4).

**Table 4:** Phenolic compounds present in different extracts of *Termitomyces* species.

*Termitomyces* Species	Solvent Extraction	Phenolic Compounds	References
*T. clypeatus*	Ethanol	Pyrogallol and Cinnamic acid	[206,207]
	Methanol	p-Coumaric acid, Ferulic acid, Gallic acid, p-Hydroxybenzoic acid, and Myricetin	[205]
*T. heimii*	Methanol	Caffeic acid, Cinnamic acid, Coumaric acid, Gallic acid, Gentisic acid, Protocatechuic acid, Pyrogallol, Tannic acid, and Vanillic acid	[204]
	Water	Cinnamic acid, Coumaric acid, Ferulic acid, Gallic acid, Gentisic acid, Protocatechuic acid, Tannic acid, and Vanillic acid	
	Ethanol	Cinnamic acid and Pyrogallol	[208]
*T. letestui*	Methanol	Caffeic acid, Chlorogenic acid, p-Coumaric acid, Ferulic acid, Gallic acid, p-Hydroxybenzoic acid, and Myricetin	[205]
*T. medius*	Ethanol	Pyrogallol	[209]
*T. microcarpus*	Ethanol	Cinnamic acid, Gallic acid, Myrecetin, Pyragallol, and Vanillic acid	[210,211]
	Water	Caffeic acid, Ferulic acid, Gallic acid, Gentisic acid, Protocatechuic acid, Syringic acid, and Vanillic acid	[204]
	Methanol	Caffeic acid, Ferulic acid, Gallic acid, Gentisic acid, Myricetin, p-Coumaric acid, p-Hydroxybenzoic acid, Protocatechuic acid, Tannic acid, and Vanillic acid	[204,205]
*T. mummiformis*	Water	Cinnamic acid, Gallic acid, Gentisic acid, Protocatechuic acid, Syringic acid, and Tannic acid	[204]
	Methanol	Cinnamic acid, Gallic acid, Gentisic acid, Protocatechuic acid, Syringic acid, and Tannic acid	
*T. robustus*	Methanol and Ethanol	Caffeic acid, Catechin, Chlorogenic acid, Ellagic acid, Epicatechin, Gallic acid, Isoquercitrin, Kaempferol, Quercetin, Quercitrin, and Rutin	[196]
*T. shimperi*	Water	Caffeic acid, Gallic acid, Gentisic acid, Protocatechuic acid, and Vanillic acid	[204]
	Methanol	Cinnamic acid, Ferulic acid, Gallic acid, Gentisic acid, Protocatechuic acid, and Tannic acid	
*T. tylerance*	Water	Caffeic acid, Gallic acid, and Protocatechuic acid	[204]
	Methanol	Caffeic acid, Gallic acid, Gentisic acid, Syringic acid, and Tannic acid	

### 7.2. Polysaccharides

Polysaccharides obtained from edible mushrooms are one of the most interesting constituents possessing a range of mediational properties, nutritional value, and antioxidant proprieties [212,213]. Several previous studies [214,215,216,217,218,219] have reported on the polysaccharides obtained from different *Termitomyces* species and also investigated their mediational and nutritional properties, as has been summarized in Table 5.

**Table 5:** Sugar compositions of polysaccharide from different *Termitomyces* species.

*Termitomyces* Species	Polysaccharide Fraction	Sugar Compositions	Medicinal Importance	References
*T. clypeatus*	Water-soluble heteroglycan	D-glucose, D-galactose, D-mannose, and L-fucose	Antioxidant properties	[216]
*T. eurhizus*	Water-soluble polysaccharide (PS I and PS II)	D-glucose	NR	[217]
*T. heimii*	Water-soluble polysaccharide (THP-I)	Glucose	Antimicrobial, Anticancer, and Antioxidant properties	[152]
*T. microcarpus*	Water soluble glucan	D-glucose	NR	[215,218]
	α-glucans (TM I) and β-glucans (TM II)	D-glucose	NR	
*T. robustus*	β-glucans (PS I and PS II)	D-Glucose	Macrophage, Splenocyte, and Thymocyte activation	[219]
	Water-soluble fucoglucan	L-fucose and D-glucose	NR	
*T. striatus*	Heteropolysaccharide (PS-I)	D-glucose, D-galactose, D-mannose and L-fucose	NR	[220]

### 7.3. Other Bioactive Components

**Figure 4:**
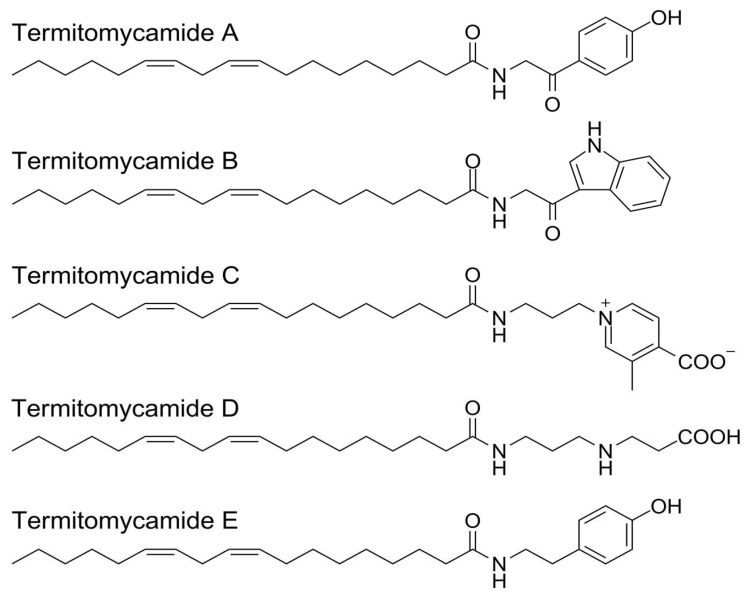
Cerebrosides isolated from *T. titanicus* (modified from Choi et al. [223]).

A huge array of bioactive compounds has been reported to originate from different *Termitomyces* species, including cerebrosides, ergostanes, fatty acid amides, serine, saponins, and protease [221,222,223,224]. Among them, cerebrosides play an important role in the treatment of several diseases. These include neurodegenerative disorders such as Alzheimer’s disease [222]. Monoglycylceramides are a group of glycosphingolipids commonly known as cerebrosides. To date, different cerebrosides (Termitomycamides A to E) have been extracted from *T. titanicus* [223] (Figure 4). Termitomycamide B and E showed the protective activity against endoplasmic reticulum stress-dependent cell death. Fatty amides include nitrogen derivative fatty acids, alcohol, or olefines obtained from natural sources or petrochemical raw materials [225]. Fatty acid amines have great industrial potential to be used in many fields including water treatment, agrochemical production, personal care, fabric softeners, paints, and coatings [225]. Importantly, five fatty acid amides have been isolated from *T. titanicus* [223]. 

## 8. Research on Antioxidant Activity

Free radicals are produced from molecular oxygen via various endogenous processes (physiological and metabolic processes) and from a variety of exogenous sources (ionizing radiation, ultraviolet light, and various pollutants). They are generally referred to as reactive oxygen species (ROS) [226,227,228]. The production of free radicals can have a negative effect on the state of health of living organisms including humans [227,228,229]. All organisms can protect themselves from different free radical damage that is induced by oxidative enzymes (catalase, superoxide dismutase, and peroxidase) and chemical compounds (*α*-tocopherol, ascorbic acid, carotenoids, and glutathione) [230] due to their antioxidant activity. When the mechanism of antioxidant fortification becomes disturbed via free radical activity, it can lead to several diseases such as arteriosclerosis, cancer, cirrhosis, and rheumatoid arthritis, as well as certain degenerative processes associated with aging [227,228,229].

The in vitro antioxidant activity of different *Termitomyces* species has been well investigated in different countries across Asia and Africa. Different extracts obtained from various *Termitomyces* species can exhibit beneficial antioxidant proprieties. The methanolic extracts of *T. eurrhizus* [231,232], *T. heimii* [233,234], *T. microcarpus* [204,233], *T. mummiformis* [204], *T. robustus* [232,235], *T. sagittiformis* [236], and *T. schimperi* [204] have exhibited beneficial antioxidant activity. Another extraction procedure using ethanol revealed significant activity via different antioxidant screening methods on *T. microcarpus* [210], *T. heimii* [208], *T. medius* [237], *T. clypeatus* [154], *T. eurrhizus* [231,232], and *T. reticulatus* [238]. Polysaccharides obtained from (crude or purified) *Termitomyces* have been determined to be efficient in reducing ROS and have exhibited effective antioxidant activity. Examples of this include the heteroglycan of *T. clypeatus* [216] and crud polysaccharides of *T. medius* [239]. However, many other extraction procedures employing water and/or chloroform have also been used to evaluate the antioxidant activity of different *Termitomyces* species [154,204,231,232]. Some examples of antioxidant activity of *Termitomyces* species are shown in the Table 6.

**Table 6:** In vitro antioxidant activity from different *Termitomyces* species.

*Termitomyces* Species	Sample Type/Solvent Extraction or Fraction	Antioxidant Activity [EC_50_ or IC_50_ (µg/mL)]	References
*T. clypeatus*	Fruitbody/Water soluble heteroglycan	CFI (462.10), RP (260), and SOD (180)	[216]
	Mycelia/Water	CFI (3060), DPPH (27.59), OH (21.94), NO (169.92), SOD (91.55), and TAC (64.36)	[154]
	Mycelia/Ethanol	CFI (4486.66), DPPH (86.84), NO (247.38), OH (40.67), SOD (133.08), and TAC (70.57)	
	Fruitbody/Ethanol	CFI (210), DPPH (3220), RP (1770), SOD (330), and TAC (1300)	[206]
*T. eurrhizus*	Fruitbody/Ethanol	ABTS (185.19), CFI (1533.70) DPPH (387.89–712.76), and HFRSA (357.4)	[231,232]
	Fruitbody/Water	ABTS (78.90), CFI (1046), DPPH (298.50–715.27), and HFRSA (407.50)	
	Fruitbody/Methanol	CFI (1201.90) and DPPH (171–717.65)	
*T. heimii*	Fruitbody/Phenolic	DPPH (490), OH (21), SOD (190), and RP (1310)	[240]
	Fruitbody/Ethanol	DPPH (1250) and RP (575)	[208]
	Fruitbody/Methanol	ABTS (185.26), CFI (216.50), DPPH (136.30–148.50), OH (162.47), and RP (257.70–833)	[233,234]
*T. medius*	Fruitbody/Ethanol	CFI (680), DPPH (500), RP (2050), and SOD (1400)	[237]
	Fruitbody/Crude polysaccharides	CFI (150), OH (960), RP (1950), and SOD (410)	[239]
	Fruitbody/Polyphenol	CFI (540), DPPH (600), LPA (1650), OH (19.5), RP (1550), and SOD (425)	
*T. microcarpus*	Fruitbody/Ethanol	CFI (140), DPPH (1980), RP (1650), and SOD (295)	[210]
	Fruitbody/Polyphenol	CFI (1300), DPPH (600), OH (16), RP (1700), and SOD (350)	[211]
	Fruitbody/Methanol	ABTS (206.36), CFI (240.90), DPPH (181.50–1600), OH (207.26), RP (276.24), and SOD (172.70)	[204,233]
	Fruitbody/Water	DPPH (2800)	[204]
*T. mummiformis*	Fruitbody/Water	DPPH (1180)	[204]
	Fruitbody/Methanol	DPPH (3700)	
*T. reticulatus*	Fruitbody/Ethanol	ABTS (1370), DPPH (4920), LPA (2053), and RP (2587)	[238]
*T. robustus*	Fruitbody/Methanol	DPPH (716.60–4780), LPO (430), and RP (1240)	[232,235]
	Fruitbody/Ethanol	DPPH (710)	[232]
	Fruitbody/Water	DPPH (714.93)	
*T. sagittiformis*	Fruitbody/Methanol	DPPH (27760) and FRAP (22.10)	[236]
*T. schimperi*	Fruitbody/Aqueous	DPPH (2100)	[204]
	Fruitbody/Methanol	DPPH (1800)	
*T. tylerance*	Fruitbody/Aqueous	DPPH (2760)	[204]
	Fruitbody/Methanol	DPPH (1240)	

Chelating ability of ferrous ions = CFI; Superoxide radical scavenging = SOD; DPPH radical scavenging activity = DPPH; Hydroxyl radical scavenging effect = OH; NO radical scavenging effect = NO; Total antioxidant activity = TAC; ABTS free radical scavenging activity = ABTS; H_2_O_2_ free radical scavenging activity = HFRSA; Lipid peroxidation activity = LPA; Ferric-reducing antioxidant power = FRAP; Reducing power assay = RP; Half maximal effective concentration = EC_50_; Half-maximal inhibitory concentration = IC_50_.

## 9. Research on Antimicrobial Activity

Presently, modern healthcare practices face a significant challenge in their battle against microbial drug-resistance as many antimicrobial agents are losing their efficacy. For example, cephalosporin and quinolones (*β*-lactam antibiotics) are routinely being used to treat *E. coli* infection, but currently they have begun to lose their effect [241,242]. The importance of developing alternative therapies and agents against drug resistant bacteria, as well as other potentially dangerous micro-organisms, have also been indicated by the World Health Organization [243]. Many antibiotics have been derived from natural sources and these have been developed as safe supplements in the administration of antimicrobial therapies [244]. Wild edible mushrooms contain a wide range of low- and high-molecular weighted compounds that could be developed as safe and natural sources of antibiotics. Several reports have indicated that macro fungi possess good antimicrobial properties that can further be employed in the pharma industry [6,245,246]. Members of the genus *Termitomyces* have shown significant results in reacting against various human pathogenic bacteria and some fungal pathogens, but no studies have yet been undertaken involving other microorganisms [133]. Among the various *Termitomyces* species, *T. clypeatus*, *T. eurrhizus*, *T. heimii*, and *T. robustus* showed significant antimicrobial activity against different pathogenic microorganisms [234,247,248,249,250,251]. Some polysaccharides (endo- and exo-polysaccharides) from *T. heimii* also showed significance antimicrobial activity against different micro-organisms [152,251]. The antimicrobial activities of different *Tremitomyces* species have been summarized in Table 7. 

**Table 7:** Different *Termitomyces* species with antimicrobial activities.

*Termitomyces* Species	Solvent Extraction	Inhibition Growth of Microorganism	Experimental Method	References
*T. clypeatus*	Water	Candida albicans, C. glabrata, Enterobacter aerogenes, Escherichi coli, Salmonella typhi, and Staphylococcus aureus	Disc diffusion	[247,248]
	Methanol	Pseudomonas aeruginosa		
*T. eurhizus*	Methanol	Escherichia coli and Proteus vulgaris	Disc diffusion	[231,247]
	Water	Bacillus brevis and Vibrio cholera	Disc diffusion	
	Ethanol	B. brevis and V. cholera	Disc diffusion	
	Methanol	V. cholera	Disc diffusion	
*T. heimii*	Water	Escherichia coli, Klebsiella pneumoniae, Pseudomonas sp., Staphylococcus aureus, and Streptococcus pyogenes	Well diffusion	[234,251]
	Water	Escherichia coli, Ralstonia sp., Salmonella sp., Staphylococcus aureus, and Streptococcus sp.	Disc diffusion	
*T. letestui*	Water	Escherichia coli, Salmonella typhi, and Staphylococcus aureus	Disc diffusion	[249]
*T. microcarpus*	Methanol	Bacillus cereus and Proteus vulgaris	Disc diffusion	[247]
*T. robustus*	Ethanol	Candida troplicalis, Escherichia coli, and Shigella dysenteriae	Well diffusion	[196,250]
	Ethanol	Aspegillus fumigatus, Staphylococcus aureus and Trichoderma rubum	Well diffusion	
	Methanol	Staphylococcus aureus	Well diffusion	
*Termitomyces* sp.	Chloroform	Candida albicans, C. parapsilosis, Escherichia coli, Klebsiella pneumonia, Pseudomonas aeruginosa, and Staphylococcus aureus	Well diffusion	[252]
	Ethanol	Candida albicans, C. parapsilosis, Escherichia coli, Klebsiella pneumoniae, Pseudomonas aeruginosa, and Staphylococcus aureus	Well diffusion	
	Water	Candida albicans, C. parapsilosis, Escherichia coli, Klebsiella pneumoniae, Pseudomonas aeruginosa, and Staphylococcus aureus	Well diffusion	
*T. striatus*	Dichloromethane	Bacillus subtilis, Escherichia coli, Pseudomonas aeruginosa, and Staphylococcus aureus	Disc diffusion	[133]
	Methanol	Bacillus subtilis, Escherichia coli, Pseudomonas aeruginosa, and Staphylococcus aureus	Disc diffusion	
	Water	Bacillus subtilis, Escherichia coli, Pseudomonas aeruginosa, and Staphylococcus aureus	Disc diffusion	

## 10. Research on Human Diseases 

### 10.1. Research on Anticancer Activity 

**Figure 5:**
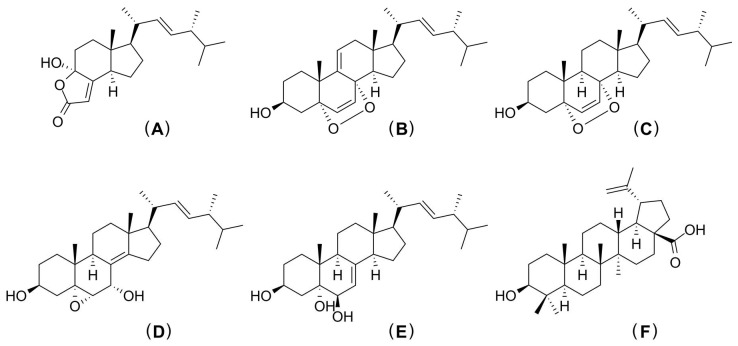
Anticancer compounds isolated from *T. microcarpus* (modified from Njue et al. [224]). Dimethylincisterol (**A**), 5*α*,8*α*–epidioxy-(22*E*,24*R*)-ergosta-6,9(11),22-trien-3*β*-ol (**B**), 5*α*,8*α*–epidioxy-(22*E*,24*R*)-ergosta-6,22-dien-3*β*-ol (**C**), 5*α*,6*α*-epoxy-(22*E*,24*R*)-ergosta-8(14),22-diene-3*β*,7*α*-diol (**D**), (22*E*,24*R*)-ergosta-7,22-diene-3*β*,5*α*,6*β*-triol (**E**) and betulinic acid (**F**).

Presently, cancer therapies, e.g., radiotherapy and chemotherapy, can have a variety of effects on the immune system [253]. Immunomodulatory agents derived from biological sources have received attention for their minimal or non-existent side effects on the human immune system. Among them, mushrooms may be a great alternative source in the development of effective cancer treatments [254,255]. However, the mechanism of the immunomodulatory effect of mushroom polysaccharides is not yet clear. Generally, mushroom polysaccharides do not assert cytotoxic effects on tumor cells but can enhance an immunomodulatory response [256]. Many recent studies have claimed that mushrooms have potential to be used in the development of cancer therapies [255,257,258,259]. *Termitomyces* have not yet been fully investigated for use in the development of cancer treatments when compared to other edible mushrooms. However, a few studies involving the *Termitomyces* species viz. in vivo study of water-soluble crude polysaccharides of *T. heimii* have indicated an effective decrease in hyperplasia on colon cancer in Swiss albino rats when induced by 1, 2-dimethylhydrazine. Consequently, they could be used in the development of treatments for other forms of cancer [152]. In this regard, *T. schimperi* combined with kaolin has exhibited mutagenic potential [153]. Ergostane is a steroid hydrocarbon that has strong potential to be used in the development of new therapeutics in the treatment of a number of diseases (e.g., several types of cancer). Very limited research has been conducted on ergostane obtained from *Termitomyces* to date. Njue et al. [224] isolated five types of ergostane (namely dimethylincisterol, 5*α*,8*α*–epidioxy-(22*E*,24*R*)-ergosta-6,9(11),22-trien-3*β*-ol, 5*α*,8*α*–epidioxy-(22*E*,24*R*)-ergosta-6,22-dien-3*β*-ol, 5*α*,6*α*-epoxy-(22*E*,24*R*)-ergosta-8(14),22-diene-3*β*,7*α*-diol and (22*E*,24*R*)-ergosta-7,22-diene-3*β*,5*α*,6*β*-triol) and betulinic acid (Figure 5) from *T*. *microcarpus* that exhibited potential against cancer with leukemia SR line, the melanoma LOX IMVI line, the breast cancer cell line T-47D, colon cancer cell lines, as well as some ovarian, prostate, and CNS cancer cell lines. The aqueous extract of *T. clypeatus* displayed cytotoxicity against several cell lines (U373MG, MDA-MB-468, HepG2, HL-60, A549, U937, OAW-42, and Y-79). However, it exhibited higher activity against the cell line U937 and significantly decreased tumors, while increasing hemoglobin and RBC counts, and increasing the mean survival time of all subjects [154].

### 10.2. Research on Other Human Diseases

According to Anchang et al. [260], *T. titanicus* was capable of increasing hemoglobin levels (12.2 g/dl) and white blood cells (26300 cells/mm^3^) when compared to treatments involving vitamin B complex on albino rat models; however, they may also be used in the treatment of Noma disease (cancrum oris) A preliminary study of polysaccharide *T. eurhizus* exhibited antiulcerogenic properties in mice models. This could be useful in the treatment of gastroduodenal diseases that are caused by non-steroidal anti-inflammatory drugs [214]. A water-soluble polysaccharide fraction obtained from *T. eurrhizus* was found to dose-dependently inhibit the replication of intracellular amastigotes of *Leishmania donovani* in macrophages [261]. 

On the other hand, some previous studies reported that *Termitomyces* contains alpha-emitting radioisotopes (^137^Cs, ^40^K, ^226^Ra, ^232^Th, and ^235^U) and may have negative effects on human health [262,263]. Notably, *Termitomyces* also accumulate various amounts of arsenic (As) which is a significant risk to human health when consumed [264].

## 11. Enzymes for Industrial Implementation

There has been a recent trend toward employing biological processes over chemical processes for industrial applications in order to reduce the resulting amounts of environmental pollution, wherein fungal enzymes can play an important role in the textile, leather, paper, and pulp industries, and particularly in the food industry [265,266,267,268]. For example, Xylanase can be produced by a large number of fungal genera, including *Aspergillus*, *Fusarium*, *Penicillium*, *Pichia*, and *Trichoderma*, and is widely used in the production of biofuels, in the food production industry, as well as in the paper and pharmaceutical industries [267,268]. However, very few reports have been made available involving the genus *Termitomyces* that establish whether it can be used in industrial applications, whereas research on the enzyme production of *Termitomyces* could be widely used for various industrial purposes. Accordingly, Majumder et al. [265] reported on metalloprotease (κ-casein specific) obtained from *T. clypeatus*, which is a new source of milk-clotting protease that can be used as a substitute for chymosin in cheese production. Another report on the same species has confirmed that it produced extracellular alkaline protease, which could efficiently depilate goat skin and separate bird feather vanes from the shaft [268]. Many other *Termitomyces* species that produce a wide range of lignocellulolytic enzymes have been summarized in Table 8. These lignocellulolytic enzymes can potentially be used in a number of important industries.

**Table 8:** Enzyme isolated from different *Termitomyces* species.

*Termitomyces* Species	Enzyme	References
*T. clypeatus*	Lignocellulases	[269]
	Carboxymethyl Cellulase	[270]
	Xylanase	
	Cellobiose Dehydrogenase	[271]
*T. eurrhizus*	α-galactosidase	[272]
*T. heimii*	Lignocellulases	[273]
*Termitomyces* sp. OE147	Cellobiose Dehydrogenase	[274]

## 12. Future Prospects and Conclusions

Currently, mushrooms and natural compounds derived from mushrooms have become a popular supplementary food and have been recognized as a potential health promoter. However, at present, many ongoing research studies have focused on the industrial development of wild edible mushrooms and their cultivation. Artificial cultivation techniques of wild edible mushrooms, especially *Termitomyces*, have not yet been available to date, but several researchers have been attempting to develop artificial techniques for the cultivation and mass production of termite mushrooms. The taxonomic implementation of *Termitomyces* is based on multi-gene phylogenetic concepts employed in conjunction with detailed morphology. The *Termitomyces* species are known to possess several nutritional and mediational prospects that involve a wide array of secondary metabolites, vitamins, and micro-nutrients. These are known to possess beneficial antimicrobial, anticancer, and antioxidant properties, indicating that they can possibly be a source in future drug development efforts. *Termitomyces* can be used in the food industry, while different enzymes derived from *Termitomyces* can be used in several industrial applications including those of the textile, leather, paper, and pulp industries. The ethno-medicinal importance of this genus needs to be further explored in terms of its prominence in various ethnic communities. Moreover, the traditional knowledge of this species that can be obtained from local communities in different regions may play a significant role in contributing to modern medical research, which may help researchers discover alternative natural sources for use in antibiotic development. 

## Data Availability

Not applicable.
